# Combined inhibition of EGFR and c-ABL suppresses the growth of triple-negative breast cancer growth through inhibition of *HOTAIR*

**DOI:** 10.18632/oncotarget.3441

**Published:** 2015-03-10

**Authors:** Yuan-Liang Wang, Anne-Marie Overstreet, Min-Shan Chen, Jiang Wang, Hua-Jun Zhao, Po-Chun Ho, Molly Smith, Shao-Chun Wang

**Affiliations:** ^1^ Department of Cancer Biology, University of Cincinnati College of Medicine, Cincinnati, OH, USA; ^2^ Cancer and cell biology graduate program, University of Cincinnati, Cincinnati, OH, USA; ^3^ Department of Pathology & Lab Medicine, University of Cincinnati College of Medicine, Cincinnati, OH, USA; ^4^ School of Pharmacy, Zhejiang Chinese Medical University, Zhejiang, China

**Keywords:** EGFR, c-ABL, lncRNA, b-catenin, breast cancer

## Abstract

Triple-negative breast cancer (TNBC) does not express conventional therapeutic targets and is the only type of malignant breast cancer for which no designated FDA-approved targeted therapy is available. Although overexpression of epidermal growth factor receptor (EGFR) is frequently found in TNBC, the therapeutic effect of EGFR inhibitors in TNBC has been underwhelming. Here we show that co-treatment with clinically validated inhibitors of c-ABL (imatinib) and EGFR (lapatinib) results in synergistic growth inhibition in TNBC cells. The dual treatment leads to synergistic repression of the long non-coding RNA (lncRNA) *HOTAIR* (*HOX Antisense Intergenic RNA*). *HOTAIR* has been known to induce tumor growth and metastasis in breast cancer. Depleting *HOTAIR* alone phenocopies the dual treatment in growth suppression. We show that expression of *HOTAIR* is regulated by β-catenin through a LEF1/TCF4-binding site. The dual treatment blocks nuclear expression of β-catenin and prevents its recruitment to the *HOTAIR* promoter. Consistently, forced expression of β-catenin rescued *HOTAIR* expression and cell viability in the presence of both drugs. Upregulation of *HOTAIR* is associated with TNBC in cell lines and a cohort of primary tumors. This study elucidates a previously unidentified mechanism in TNBC linking signaling with lncRNA regulation which may be exploited for therapeutic gain.

## INTRODUCTION

Triple-negative breast cancer (TNBC), accounting for 10%–20% of breast cancer, is characterized by the lack of estrogen receptor (ER), and progesterone receptor (PgR), and low expression of the receptor tyrosine kinase ErbB2 (also known as HER2/*neu*). TNBC are often highly proliferative, of higher grade, and tend to be more aggressive than other types of breast cancer [1]. Since TNBC is devoid of conventional therapeutic targets, it is the only major breast cancer type for which no specific FDA-approved targeted therapy is available. Radio- and chemo-therapies are the treatment options for TNBC. Thus, efficient targeted therapeutic regimens are urgently needed for TNBC.

EGFR is a receptor tyrosine kinase (RTK) of the ErbB family [2]. Multiple signaling pathways, such as PI3K/AKT, mitogen-activated protein kinase (MAPK), and Wnt/β-catenin are activated by EGFR to enhance proliferation, survival, invasion, and metastasis of cancer cells [3]. Expression of EGFR is frequently associated with TNBC and has been viewed as a promising therapeutic target [4–7]. Unfortunately, the therapeutic efficacy of EGFR-targeting agents has been disappointing in breast cancer [8, 9], suggesting that other molecular drivers also contribute to the malignancy. The non-receptor tyrosine kinase c-ABL promotes cell proliferation, migration, and survival [10]. Multiple studies including ours have demonstrated the important role of the non-receptor tyrosine kinase c-ABL in breast cancer [11–16]. We also showed that c-ABL expression is a frequent event in breast cancer and is associated with advanced tumor stages and metastasis [15, 16]. Our recent report showed that combined treatment with lapatinib, a dual inhibitor of EGFR and ErbB2/HER2, and imatinib, a c-ABL inhibitor, resulted in synergistic growth inhibition in a panel of EGFR/ErbB2-expressing breast cancer cells including the TNBC cell line MDA-MB-468 [17]. While this observation suggests the potential of combined inhibition of EGFR and c-ABL in targeting TNBC, the underlying mechanism awaits to be determined.

Long non-coding RNAs (lncRNAs) are non-coding RNAs greater than 200nt in length. Emerging evidence demonstrates the fundamental functions of lncRNAs in regulating genes associated with human diseases, including cancer [18, 19]. *HOTAIR* (*HOX antisense intergenic RNA*) is a 2.3 kb non-coding transcript derived from the intergenic region of the *HOXC* homeotic gene cluster. It functions as a scaffold to assemble epigenetic moderators to regulate gene expression [20]. *HOTAIR* was the first lncRNA shown to promote tumor progression and is associated with poor prognosis in breast cancer [21]. Expression of *HOTAIR* enhanced the growth and metastasis of xenograft tumors of mammary fat pad [21]. However, virtually nothing is known about how this important lncRNA is regulated in cancer cells, or whether targeted therapeutic drugs affect its expression.

Here we report that the combined treatment is an effective approach to inhibit the growth of multiple TNBC cell lines, and identify *HOTAIR* as a downstream gene. We demonstrate that *HOTAIR* expression is transcriptionally repressed by the combined treatment of lapatinib plus imatinib through inhibition of β-catenin. We further show that *HOTAIR* expression is closely correlated with primary TNBC tumor tissues.

## RESULTS

**Figure 1:**
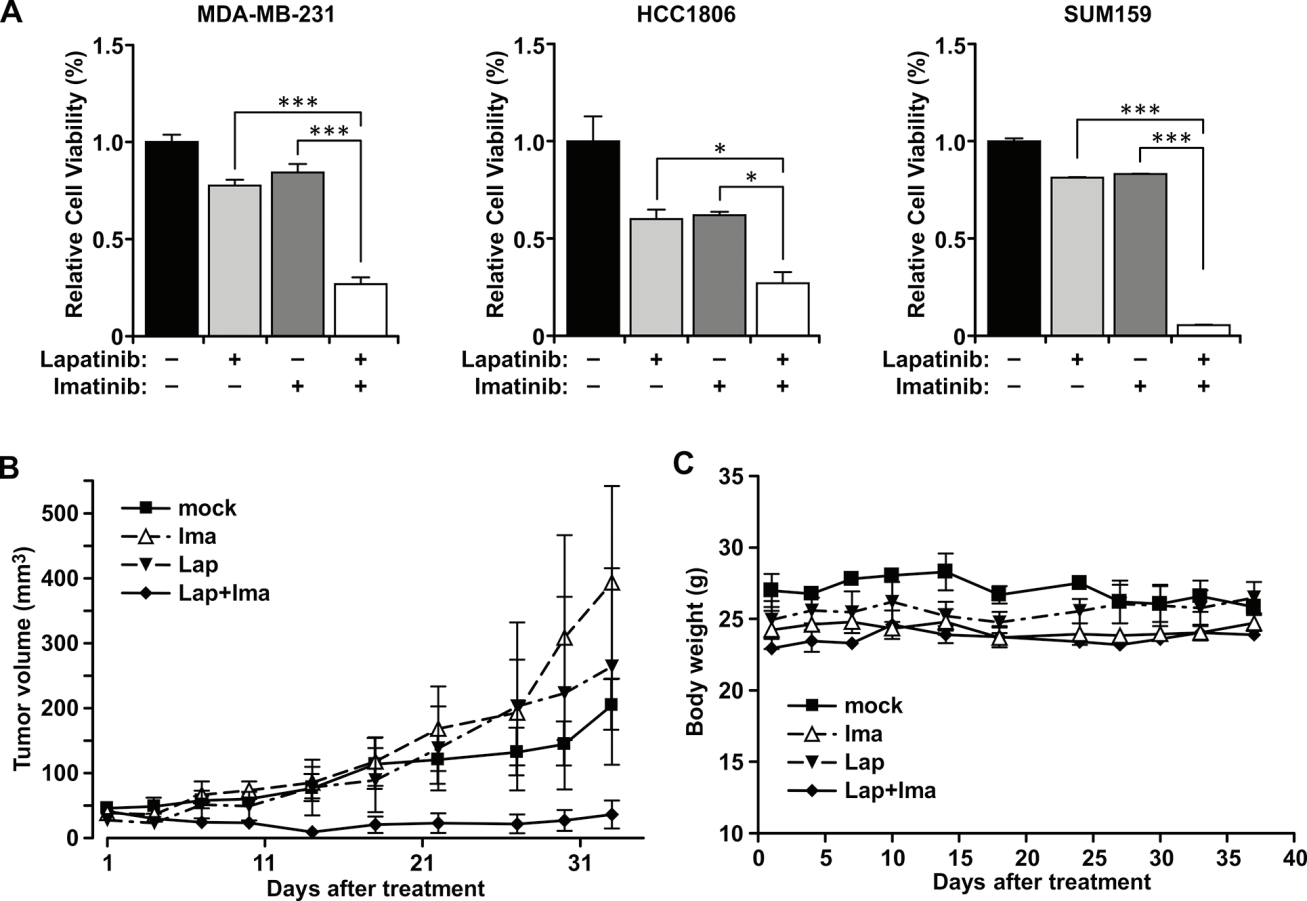
Combination treatment with lapatinib and imatinib inhibited growth of cells and tumors **(A)** MDA-MB-231, HCC1806, and SUM159 cells were inoculated in 96-well plates and mock treated or treated with the drugs for 72 hrs. MDA-MB-231 and HCC1806 cells were mock treated (without the drugs), or treated with individual drugs (10 μM) or the combination. SUM159 cells, which are more sensitive to lapatinib than other cell lines, were mock treated, or treated with individual drugs (5 μM) or their combination for 72 hours. Cell growth was evaluated by MTT assay. *, *P* < 0.05, ***, *P* < 0.005. **(B)** MDA-MB-231 cells (1 × 10^7^) were inoculated in the mammary fat pads of female nude mice. Tumor-bearing mice were mock-treated, or treated with lapatinib alone (100 mg/kg), imatinib alone (100 mg/kg), or both. Tumor volumes were plotted. Bars, standard deviation. Statistical significance is shown in Supplementary Table S1. **(C)** The body weights of the same mice under the indicated treatments in B were monitored throughout the study. Bars, standard deviation.

We first tested that the growth inhibition effect of combined treatment with lapatinib and imatinib in MDA-MB-231 cells which, like MDA-MB-468, do not express estrogen receptors or ErbB2/HER2. Although treatments with lapatinib and imatinib effectively inhibit the activity of EGFR and c-ABL respectively (Supplementary Figure S1A), the treatments with each agent alone are significantly less effective in inhibiting cell growth than the combined treatment (Figure 1A). The synergism of combining both drugs is tested by an isobologram analysis which indicates that inhibition of lapatinib and imatinib is synergistic (Supplementary Figure S1B). This is consistent to our previous observation on MDA-MB-468 cells [17], and can be extended to other TNBC cell lines including HCC1806 and SUM159. It has been shown that c-ABL kinase is the major target of imatinib in breast cancer cells [12, 13]. Together, these results suggest that combined inhibition of EGFR and c-ABL is an effective treatment *in vitro* in a panel of TNBC cell lines.

To test whether this synergistic growth inhibition can be recapitulated *in vivo*, MDA-MB-231 cells were implanted into the mammary fat pad of female nude mice. The orthotopic xenograft tumors were then treated by gavage with each of the individual drugs, the combination of the two drugs, or the control vehicle (Figure 1B). Consistent with the *in vitro* result, while there is no significant difference among the single and control treatments, the combination treatment effectively suppressed tumor growth (Figure 1B; Supplementary Table S1). Combined administration of lapatinib and imatinib was well-tolerated in mice, as the body weights were maintained stable throughout the treatment course (Figure 1C).

**Figure 2:**
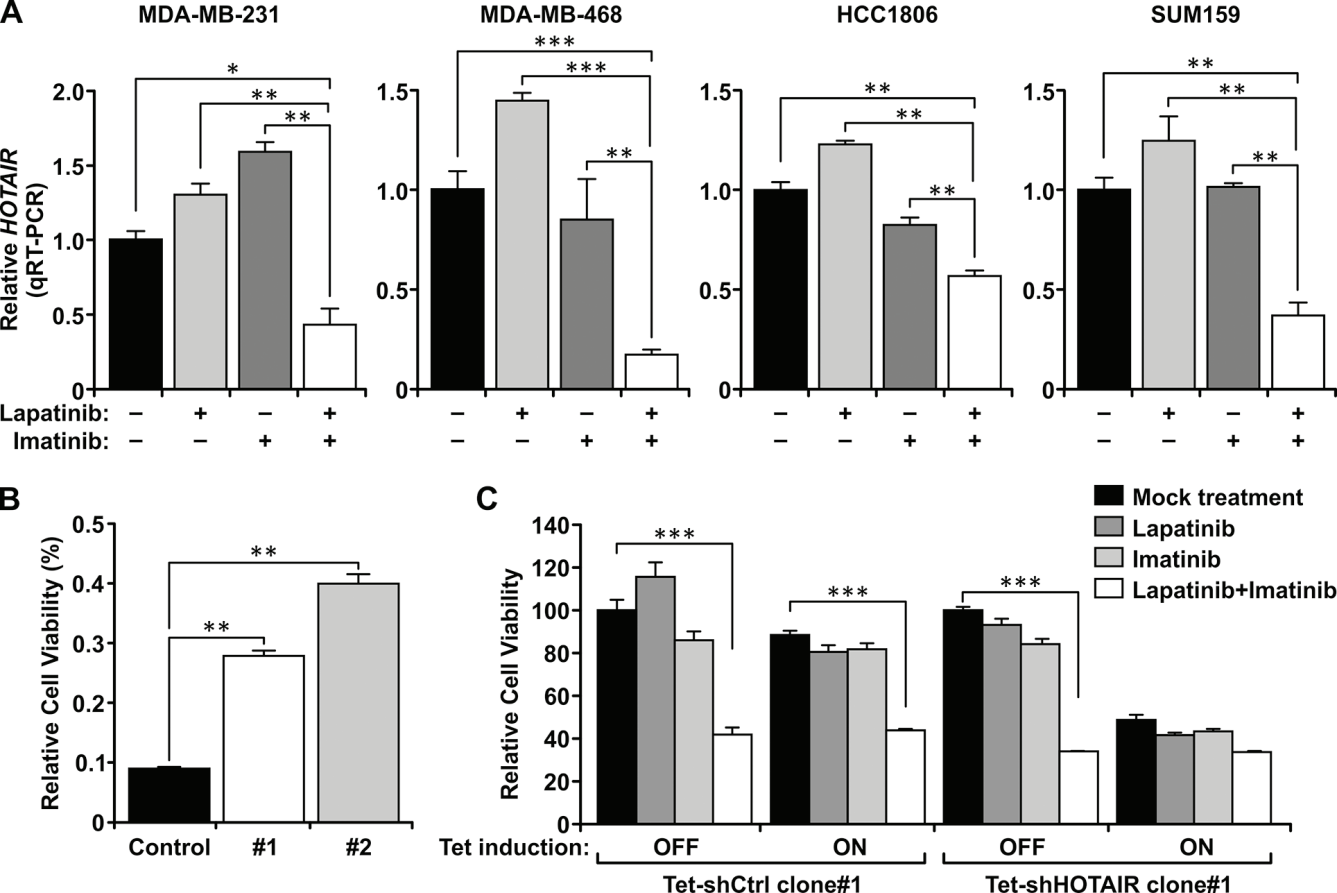
Dual treatment by lapatinib plus imatinib represses *HOTAIR* expression in TNBC cells **(A)** MDA-MB-231, MDA-MB-468, and SUM159 cells were mock treated, or treated with individual drugs (10 μM) or the combination for 48 hrs. HCC1806 cells were mock treated or treated with lapatinib alone (5 μM), imatinib alone (10 μM), or their combination (i.e. 5 μM lapatinib + 10 μM imatinib) for 48 hours. Total RNA was extracted from the cells and converted to cDNA, then subject to quantitative RT-PCR (qRT-PCR) to measure its relatively level after normalized to 18S. **(B)** MDA-MB-231 cells were stably transfected with *HOTAIR* cDNA, and two resulted independent clones together with a vector alone control transfectant line were tested. Cells were treated with and without the combination treatment (apatinib+imatinib,10 μM each). Cell growth was measured by MTT assay and the relative level of viable cells were plotted. **(C)** MDA-MB-231 cells were engineered to expression shRNA of *HOTAIR* or the control shRNA of scrambled sequence under the control of the tetracycline-inducible promoter. Cells were induced by tetracycline for 48 hrs then treated with the indicated drugs for 48 hrs. Viable cells were then measured by MTT assays. ***, P < 0.005.

**Figure 3:**
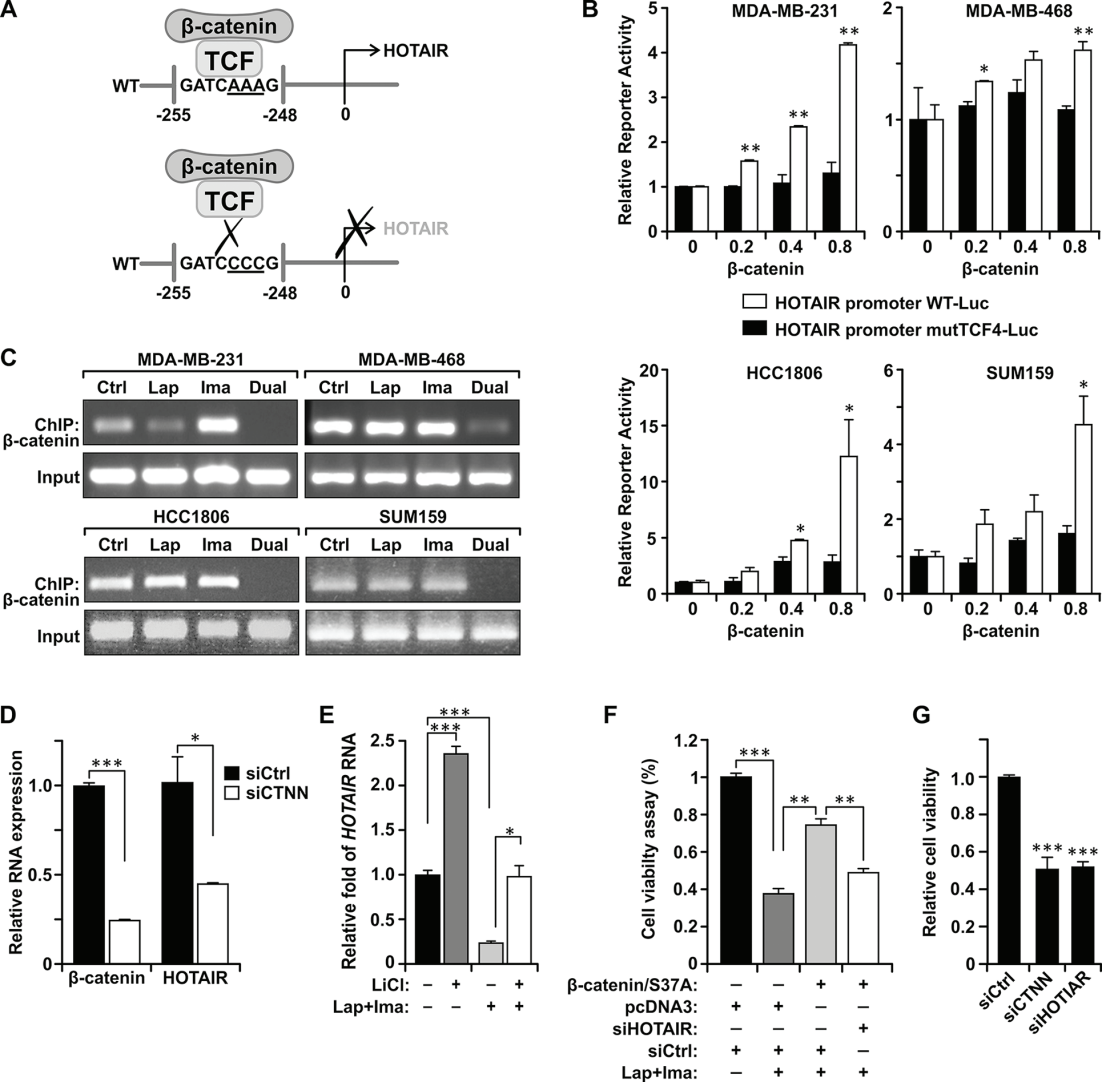
β-catenin stimulates the expression of *HOTAIR* through a TCF4/LEF1-binding site and is a down-stream target of the dual treatment **(A)** A schematic model of the identified TCF4/LEF1-binding site on the *HOTAIR* promoter. **(B)** Transient co-transfection of MDA-MB-231 cells with the luciferase reporter gene under the control of WT or mutant *HOTAIR* promoter in which the TCF4/LEF1-binding site is mutated, with and without the cDNA of β-catenin/S37A. Expression of the reporter was stimulated by the wild-type but not the mutated promoter in the indicated TNBC cell lines in a dose-dependent manner. **(C)** MDA-MB-231 cells were transfected with control siRNA (siCtrl) or siRNA against β-catenin (siCTNN). 48 hrs after transfection, RNA was isolated and the level of *HOTAIR* and β-catenin RNA were determined by qRT-PCR. *18S* RNA was used as normalization control. *, *P* < 0.05; ***, *P* < 0.005 **(D)** Dual treatment preferentially suppresses recruitment of β-catenin to the TCF4/LEF1 site of the *HOTAIR* promoter in the indicated TNBC cells. Cells were treated as described in Figure 2. The β-catenin-DNA complexes were pulled down by immunoprecipitation and the DNA fragments were subject to PCR amplification using primers flanking the TCF4/LEF1 site of the *HOTAIR* promoter.**(E)** MDA-MB-231 cells were treated with and without the combination of lapatinib and imatinib (10 μM each) for 48 hrs followed by LiCl induction for 48 hrs. *HOTAIR* expression was then determined by qRT-PCR. **(F)** The combined treatment (Lap+Ima) inhibited growth of MDA-MB-231 cells, which was rescued by ectopic expression of the constitutively active mutant β-catenin/S37A. Depleting *HOTAIR* by siRNA (siHOTAIR), but not by the control shRNA (shCtrl) of scrambled sequence, re-sensitized cells to the combined treatment. Cell viability was determined by measuring the activity of a co-transfected pcDNA3-luciferase reporter. **, *P* < 0.01; ***, *P* < 0.005. **(G)** Down-regulation of β-catenin resulted in cell growth inhibition to a similar level achieved by down-regulation of *HOTAIR*. MDA-MB-231 cells were transfected by specific siRNA of β-catenin (siCTNN) or *HOTAIR* (siHOTAIR). After 48 hours, viable cells were assessed by MTT assay. ***, *P* < 0.005 (in comparison with siCtrl).

To gain insight of the underlying mechanisms and to test the potential role of long non-coding RNA regulation in the enhanced tumor suppression activity of the dual treatment, we screened a quantitative PCR (qPCR) array of 90 lncRNAs involved in cancer and are well-documented in the lncRNA database (SBI; see Materials and Methods) [22]. Among them, the *HOTAIR* lncRNA stands out as its expression is diminished by the dual treatment but not the individual drugs (data not shown). This primary result was further confirmed in four TNBC lines (MDA-MB-231, MDA-MB-468, HCC1806, and SUM159) (Figure 2A). In each case, expression of *HOTAIR* is down-regulated only when the cells are treated with the dual treatment, but not either drug alone. This effect on *HOTAIR* is specific as the expression of other lncRNAs such as *BC200* and *MALAT-1* is not repressed by the combined treatment (Supplementary Figure S2). We then generated stable clones derived from MDA-MB-231 which express ectopic *HOTAIR* driven by the heterologous cytomegalovirus (CMV) promoter (Supplementary Figure S3). Enforced expression of *HOTAIR* confers increased resistance to the dual treatment (Figure 2B). To further test the importance of *HOTAIR* in the sensitivity to the dual treatment, we have generated MDA-MB-231 derivatives which express tetracycline-inducible shRNA of *HOTAIR* (tet-shHOTAIR) or the control shRNA of a scrambled sequence (shCtrl). Induction of *shHOTAIR* results in significant depletion of the endogenous *HOTAIR* transcript (Supplementary Figure S4). In the absence of tetracycline, cells respond to the treatments as the parental MDA-MB-231 cells. However, induction with tetracycline significantly inhibits the growth of cells harboring tet-shHOTAIR. Treatment with the combination of lapatinib and imatinib do not further inhibit their growth (Figure 2C). In contrast, cells harboring tet-shCtrl still respond to the treatments similar to the parental cells even in the presence of tetracycline. Thus, *HOTAIR* is a critical target of combined inhibition of EGFR and c-ABL. Similar results were obtained using independent clones (Supplementary Figure S5). These results for the first time unravel the role of lncRNA in the responsiveness of cancer cells to specific targeted therapeutic agents. The identification of *HOTAIR* may provide a clue to further understanding how lncRNAs regulate the responsiveness of cancer cells in response to cancer treatments. To this aim, examining the proximal sequence of *HOTAIR* promoter (0~-1000 nt) predicted a sequence motif perfectly matched the β-catenin-TCF4/LEF1 transcription factor binding site (^−256^GATCAAAG^−249^ [23]) (Figure 3A). To test its biological significance, we constructed luciferase reporters controlled by the wild-type *HOTAIR* promoter (wtHOTAIR-Luc) and the mutant in which the TCF4/LEF1 site sequence is altered to ^−256^GATCCCCG^−249^ (mutHOTAIR-Luc). Wild-type or mutant reporter construct was then co-transfected into TNBC cells with different doses of a constitutively active hemagglutinin (HA)-tagged β-catenin/S37A mutant (serine 37 replaced with alanine; [24]) and the consequent luciferase activities were assessed (Figure 3B, Supplementary Figure S6A). The wild-type reporter is enhanced by β-catenin in a dose-dependent manner, while the mutant reporter does not respond to β-catenin-mediated transactivation. These results demonstrate that the *HOTAIR* lncRNA is regulated through the consensus β-catenin-TCF4/LEF1 site by β-catenin.

The results described above predict that β-catenin is physically recruited to the *HOTAIR* promoter at the TCF4/LEF1 site. This is supported by chromatin immunoprecipitation (ChIP) of β-catenin in which the associated genomic region spanning the TCF4/LEF1 site was amplified by primers flanking the site (Figure 3C). The experiment also demonstrates that recruitment of β-catenin to the *HOTAIR* promoter is diminished upon the dual treatment of lapatinib plus imatinib, but not either drug alone. These results are in concord with the mechanism that β-catenin promotes *HOTAIR* expression which is the main target by the dual treatment of lapatinib plus imatinib to block cell growth of TNBC. Consistently, treatment with specific β-catenin siRNA, but not the control siRNA, leads to significant down-regulation of *HOTAIR* (Figure 3D). Conversely, exposure to LiCl, which induces the classic β-catenin pathway [25, 26], stimulates *HOTAIR* expression and rescues *HOTAIR* expression in cells treated with lapatinib plus imatinib (Figure 3E). Indeed, ectopic expression of β-catenin/S37A rescues cells from the inhibition of the dual treatment, while depleting the endogenous *HOTAIR* re-sensitizes cells to the dual treatment (Figure 3F, Supplementary Figure S6B). Consistently, depleting β-catenin by transient transfection of a specific siRNA (siCTNN) resulted in growth inhibition as did siHOTAIR (Figure 3G).

**Figure 4:**
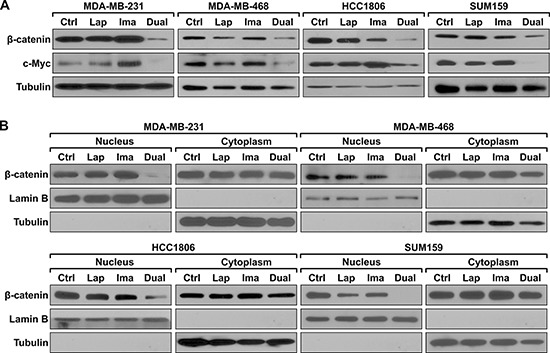
Combined treatment with lapatinib and imatinib inhibits nuclear expression of β-catenin **(A)** The indicated cell lines were treated with lapatinib, imatinib, lapatinib plus imatinib, or mock treated as described in Figure 2 with the exception of HCC1806 which was treated with 0.1 μM lapatinib, 5 μM imatinib, or the combination for 48 hrs. Total cell lysates were analyzed with the indicated antibodies. **(B)** Cells were treated as described in A except that the treatment last for 24 hrs. Cells were then subject to fractionation and the extracts from the nuclear and cytoplasmic compartments were analyzed by western blotting using the indicated antibodies. Lamin B and tubulin serve as the marker of the nuclear and cytoplasmic compartments, respectively.

To further understand the mechanism of blocking β-catenin recruitment to the *HOTAIR* promoter, protein levels of β-catenin were examined in TNBC cells subject to lapatinib and/or imatinib treatment. The dual treatment, but not the single agents, diminishes β-catenin protein expression (Figure 4A). Biochemical fractionation reveals that the dual treatment specifically down-regulates expression of nuclear β-catenin while there is no change in the cytoplasmic β-catenin levels (Figure 4B). These results establish that combined inhibition of EGFR and c-ABL inhibit the nuclear function of β-catenin. Consistently, expression of the c-Myc oncogene, which is a well-established target of β-catenin [27], is also down-regulated by the dual treatment but not the single agents (Supplementary Figure S7).

**Figure 5:**
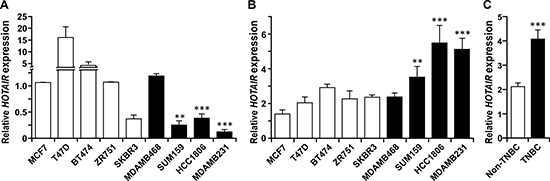
Derepression of β-catenin preferentially induces *HOTAIR* expression in TNBC cell lines **(A)** Relative expression levels of *HOTAIR* in unperturbed cells of the indicated non-TNBC (blank columns) and TNBC (black columns) cell lines. The level in MCF-7 was set as 1. Bars, standard deviations. **, *P* < 0.01; ***, *P* < 0.005 (in comparison with MCF-7). **(B)** Cells were treated with and without LiCl (40 μM) for 48 hrs, and the levels of *HOTAIR* RNA were measured by qRT-PCR. The ratios of *HOTAIR* expression in LiCl-treated and untreated cells were then determined and plotted. Three independent experiments were performed. **, *P* < 0.01; ***, *P* < 0.005 (in comparison with MCF-7). **(C)** The average fold of expression induction by LiCl in TNBC versus non-TNBC cells is shown. ***, *P* < 0.005.

**Figure 6:**
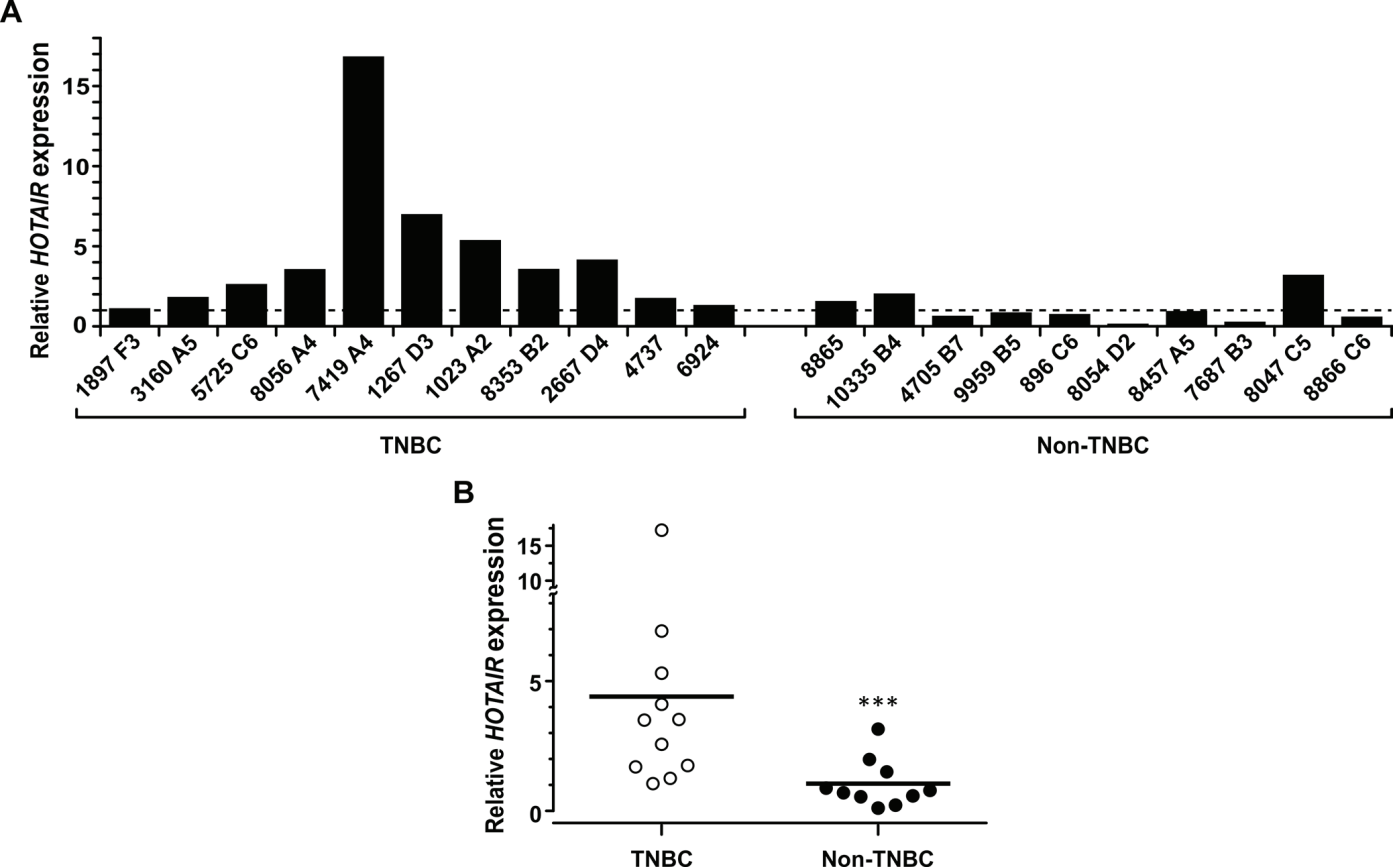
Expression of *HOTAIR* is associated with TNBC **(A)** RNA samples from primary breast cancer tumor tissues of TNBC and non-TNBC were extracted following the instruction of the manufacturer (Zymo; Irvine, CA) and the levels of *HOTAIR* were determined by qRT-PCR and normalized with actin. **(B)** The relative levels of *HOTAIR* expression in individual tissue samples was plotted and the average level of each group was indicated (bars). ***, *P* < 0.005.

Intriguingly, assessment of the steady state expression levels of *HOTAIR* in a group of TNBC and non-TNBC cell lines showed no association of *HOTAIR* expression to the triple-negative status (Figure 5A). In fact, among the tested cell lines non-TNBC cells seemed to express higher levels of *HOTAIR* than the TNBC ones. However, when comparing the extent of *HOTAIR* induction by treatment with LiCl, TNBC cells except MDA-MB-468 were significantly more sensitive to the stimulation (Figure 5B, and 5C). MDA-MB-468 cells expressed relatively high level of intrinsic *HOTAIR* compared to other cell lines (Figure 5A). This is consistent with the data that ectopic expression of β-catenin in MDA-MB-468 leads to a significant but less prominent induction of *HOTAIR* compared with other TNBC cell lines (Figure 3B). Nevertheless, these results support the essential role of β-catenin activation and enhanced *HOTAIR* expression in cultured TNBC cells. β-catenin expression is known to be a poor prognosis marker in breast cancer and its activation has been correlated with TNBC [28], further suggesting a correlation between *HOTAIR* expression and TNBC. To test this hypothesis, total RNA was extracted from a cohort of archived primary tumors (11 TNBC and 10 non-TNBC) and the level of tumoral *HOTAIR* was assessed by qRT-PCR (Figure 6A). *HOTAIR* expression in most TNBC tumors is higher than the average of *HOTAIR* expression in non-TNBC tumors, which is statistically significant (Figure 6B). Similar results were obtained using conventional semi-quantitative PCR (Supplementary Figure S8).

## DISCUSSION

**Figure 7:**
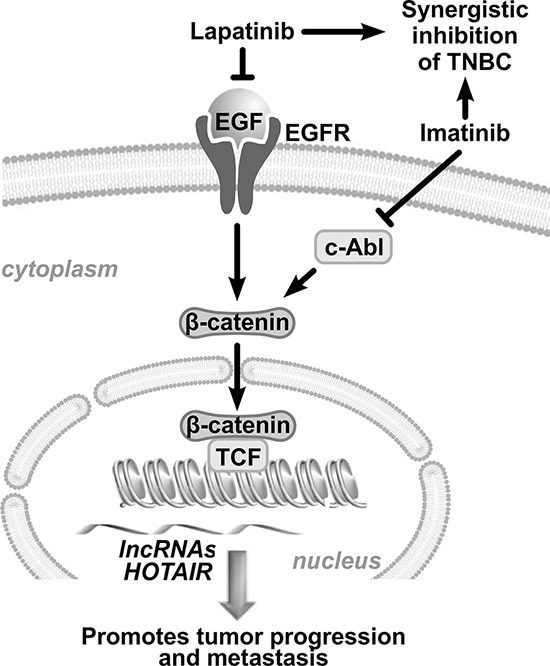
Schematic representation of the combination treatment Combined inhibition of EGFR and c-ABL leads to prominent suppression of β-catenin which transcriptionally activates the *HOTAIR* gene.

This study demonstrates the efficacy and mechanism of growth inhibition by co-targeting EGFR and c-ABL in TNBC cells (Figure 7). In consistence, c-ABL has been identified as a synthetic lethal partner of EGFR in an unbiased screening using the A431 cervical adenocarcinoma cells [29]. Both lapatinib and imatinib are FDA-approved drugs for cancer treatment. Since many FDA-approved drugs to target these two molecules have been available, the translational potential of this strategy can be realized in a relatively reasonable timeline.

Our results for the first time show that co-targeting two tyrosine kinases, but not either one alone, significantly inhibits the expression of nuclear β-catenin, suggesting that both kinases contribute to enhance β-catenin function. Multiple mechanisms of cross talk between EGFR and the Wnt pathway have been reported [30]. For example, EGFR-activated PI3K/AKT leads to phosphorylation and subsequently inhibition of the β-catenin suppressor GSK3β, resulting in nuclear accumulation of β-catenin [31]. Moreover, c-ABL has been reported to enhance nuclear translocation of β-catenin through phosphorylation of the chaperone protein p68 [32]. Phosphorylated p68 in turn facilitates β-catenin nuclear translocation by blocking phosphorylation of β-catenin by GSK-3β and displacing Axin from β-catenin [33]. EGFR is also known to promote the transcriptional transactivation activity of β-catenin by inducing Src-mediated tyrosine phosphorylation of β-catenin and its subsequent interaction with pyruvate kinase M2 (PKM2) in the nucleus [34]. Further understanding the contributions of the cross talk among the signaling pathways governed by EGFR, c-ABL, β-catenin, and *HOTAIR* is expected to shed new light to the diagnosis, treatment, and prognosis of breast cancer.

Although these results demonstrate that the combination of the clinically approved agents lapatinib and imatinib hits essential targets in TNBC cells, treatment with imatinib has been reported to promote malignant behavior of breast carcinoma cells *in vitro* [35]. This may be due to the known dual functions of c-ABL in cancer cells, as a proto-oncogene and as a check point of DNA damage response, depending on the cellular context [36–38]. While we have not observed such detrimental effect of targeting c-ABL in our system, this potential concern needs to be carefully addressed before any clinical application. In addition, to harness the maximal tumor suppression activity elicited by this treatment but avoid the complication of targeting c-ABL, it is important to identify the targets responsible for growth inhibition mediated by the combination of lapatinib and imatinib. We show that the synergistic effect of the dual treatment is mainly due to the repression of the lncRNA *HOTAIR* which is, at least in part, controlled by β-catenin through a LEF1/TCF4-binding site on the promoter. β-catenin is depleted specifically from the nucleus by the dual treatment of lapatinib and imatinib, which leads to down-regulation of *HOTAIR*. In line with this observation, depleting *HOTAIR* by RNA interference resulted in growth inhibition phenocopying the dual treatment. Thus *HOTAIR* is regulated by distinct oncogenic pathways and may be developed as a potential therapeutic target. Although the steady levels of *HOTAIR* are similar in TNBC-like and non-TNBC breast cancer cell lines, induction of β-catenin function preferentially stimulate *HOTAIR* expression in TNBC-like cell lines compared with non-TNBC cell lines. This result suggests that β-catenin's function is important in TNBC cells at least in part through the induction of *HOTAIR*. Interestingly, β-catenin expression has been associated with TNBC, but the mechanism remains unclear [28].

Our result also suggests that expression of *HOTAIR* is enriched in TNBC tumors. Although the dataset represents a limited number of patients, the results are statistically significant and consistent with the hypothesis that *HOTAIR* plays an important role in the etiology and/or pathophysiology of TNBC. This observation is contradictory to a recent report by Chisholm *et al*. which identified no correlation of *HOTAIR* with triple negative breast cancer [39]. This discrepancy could be stemmed from the different techniques entailed in these two studies. Chisholm et al. used *in situ* hybridization with synthetic RNA probes, while in the current work RNA was extracted directly from tumor tissues. In addition, the patients are derived from totally different pools in these two studies. While our observation is based on a small cohort of patients due to the low through-put nature of the technique, data presented in this report raise the possibility that *HOTAIR* plays a unique role in the etiology of at least a cohort of TNBC and warrants further investigation in larger patient populations across different disease types and stages.

We show that the dual treatment leads to down-regulation of c-Myc expression likely through the inhibition of nuclear β-catenin. c-Myc was recently identified as a transcription factor downstream of the Dicer-miR295 pathway and partly responsible for the expression of lncRNAs [40]. It remains to be determined whether c-Myc takes a part in enhancing *HOTAIR* expression as down-regulation of c-Myc by the dual treatment may further repress *HOTAIR* expression. A more recent study unveiled the Notch pathway preferentially regulating a group of lncRNAs specifically expressed in acute leukemia [41]. Whether similar mechanisms can be applied to the Wnt/β-catenin pathway in breast cancer awaits further test.

## METHODS

### Cell culture, antibodies, chemicals, and plasmids

LZRS-HOTAIR was developed in Dr. Howard Chang's laboratory [21] and purchased from Addgene (Cambridge, MA). β-catenin/S37A cDNA was a kind gift from Dr. Susan Waltz. MCF-7, T47D, BT474, MDA-MB-468, MDA-MB-231, ZR-75–1, and SK-BR3 cell lines were purchased from American Type Culture Collection (ATCC) previously. They have not been authenticated recently. SUM159 and HCC1806 were kind gifts from Dr. Sang-Oh Yoon. All cells except SUM159 and HCC1806 were grown in DMEM/F12 (1:1) with 10% fetal bovine serum. SUM159 cells were cultured in DMEM/F12 (1:1) with 5% fetal bovine serum and hydrocortisone (1 μg/ml). HCC1806 were cultured in RPMI-1640 with 1% FBS. The following antibodies were purchased: α-tubulin (Sigma); β-catenin (Bethyl), c-Myc (Pierce); lamin B (Santa Cruz), EGFR, CrkL, and phospho-Y207-CrkL (Cell Signaling). Lapatinib and imatinib were purchased from LC Laboratories. Tetracycline-inducible shRNA of *HOTAIR* and the control shRNA of scrambled sequences were constructed by annealing the corresponding oligonucleotides (100 μM for each strand denatured at 95°C for 10 min then cooled down to room temperature). The duplex inserts were amplified by PCR followed by cloning to the pCR2.1-TOPO vector (Invitrogen). The inserts were then subcloned to the pInducer-10 vector between the EcoR1 and Xho1 sites.

### Screening lncRNA library

MDA-MB-231 cells were either untreated or treated with lapatinib (10 μM), imatinib (10 μM), or the combination for 48 h. Total RNA was isolated from the cells with Trizol extraction (Invitrogen, NY). Long non-coding RNAs in the RNA samples were then profiled using a human lncRNA microarray (System Biosciences, CA; catalog# RA920D-1) following manufacturer's instructions.

### Tumor tissues

Archived formalin-fixed paraffin-embedded primary breast tumor tissues were obtained from the Department of Pathology of the University of Cincinnati. Patient identifications have been removed from the tissue samples. ER and PgR were evaluated by immunohistochemical staining (IHC). HER2 expression was based on fluorescence *in situ* hybridization (FISH) with IHC as confirmatory reference. Tissue sections of 10 μm in thickness were cut from the blocks and subject to RNA extraction using a commercial kit (Zymo Research, CA; catalog# R1007) following manufacturer's instruction.

### Cell fractionation

The cytoplasmic and nuclear fractions were prepared as described previously [42, 43]. Cultured cells were lysed in hypotonic buffer (10 mM NaCl, 10 mM Tris [pH 8], 3 mM MgCl_2_, 0.2 mM PMSF, 1 mM DTT, 0.5% Triton X-100) on ice and dounce homogenized. The nuclear pellet was washed and isolated. The nuclei were lysed in RIPA buffer (150 mM NaCl, 1% NP-40, 0.5% deoxycholate, 0.1% SDS, 50 mM Tris [pH 7.5], 25 mM NaF, 2 mM Na_3_VO_4_, 5 mM PMSF, 2 μg/ml aprotinin) and by sonication (Sonicator 4000; Misonix, Inc.).

### Western analysis

Cell lysates of the treated cells were isolated by incubation with NETN buffer (150 mM NaCl, 1 mM EDTA [pH 8.0], 20 mM Tris [pH 8.0], 0.5% NP-40, phosphatase inhibitors consisting of 25 mM NaF and 2 mM Na_3_VO_4_, and the protease inhibitors 20 μl/ml aprotinin and 0.1 M PMSF) or RIPA buffer. Cell lysates were separated on acrylamide gels, transferred to a PVDF membrane (Bio-Rad), and probed with the indicated antibodies. Bands were visualized by a chemiluminescence-based detection method (Fisher/Pierce) that used a horseradish peroxidase-conjugated secondary antibody.

### Combination indices and isobologram analysis

Data from Cell Titer-Glo assays (Promega) were analyzed using CalcuSyn statistical software (Biosoft). Isobolograms generated by CalcuSyn were based on dose-response curves for both lapatinib and imatinib. Data points below the line represent synergistic drug interactions; points on the line indicate additivity; points above the line indicate antagonism. A combination index (CI) value of one indicates additivity; a CI value greater than one indicates antagonism; and a CI value less than one indicates synergism.

### Orthotopic tumor xenografts

MDA-MB-231 cells (1 × 10^7^ in 100 μl of PBS) were mixed with equal volume of Matrigel and injected into the mammary fat pads of 8-week female nude mice. Two weeks later, when the tumor sizes reached about 50 mm^3^, mice were randomly grouped into four groups and started to be treated daily with PBS alone, lapatinib, imatinib, or the combination by oral gavage. Tumor sizes and body weights were monitored weekly.

### Chromatin immunoprecipitation

Cells were fixed with 1% formaldehyde for 10 min, and quenched in 0.125 M glycine for 5 min at room temperature. The cells were washed and lysed in cell lysis buffer (5 mM HEPES [pH 8.0], 85 mM KCl, 0.5% NP-40) at 4°C for 30 min. The nuclei were released by a Dounce homogenizer and then lysed in 100–200 μl of nuclei lysis buffer (Tris-HCl 50 mM [pH 8.0], EDTA 10 mM, SDS 1%). The lysate was sonicated on ice, and the supernatant was diluted 10-fold with dilution buffer (0.01% SDS, 1.1% Triton X-100, 1.2 mM EDTA, 16.7 mM Tris-HCl [pH 6.8], 167 mM NaCl). 1 μg of antibody was added to 1 ml of the lysate and rotated at 4°C for 2 hr. The immune-complexes were then pulled down by protein A-agarose. The beads were washed once with low salt buffer (0.1%SDS, 1%Triton X-100, 2 mM EDTA, 20mM Tris [pH 8.0], 150 mM NaCl), high salt buffer (0.1% SDS, 1% Triton X-100, 2 mM EDTA, 20 mM Tris 8.0, 500 mM NaCl), four times with Buffer III (0.25 M LiCl, 1% NP40, 1% DOC, 1 mM EDTA, 10 mM Tris [pH 8.0]), and finally once with TE. Each wash was incubated at 4°C with rotation for 10 min. The bound complexes were eluted twice with 150 μl of 1% SDS and 50 mM NaHCO_3_ each time, then added with 5 μl of 10 mg/ml RNase, 18 μl of 5M NaCl and incubated at 65°C for overnight. The reverted DNA was purified with a miniprep spin column (Qiagen) and then eluted in 50 μl of 10 mM Tris-HCl [pH 8.0].

### *HOTAIR*-Luciferase reporter and luciferase reporter assays

The *HOTAIR* promoter region (−1000~0) was amplified from the genomic DNA of MDA-MB-231 using Advantage-GC cDNA kit (Clontech) and the PCR condition of 94°C for 1 minute, 94°C for 30 seconds, 68°C 3 min for 35 cycles. Product was then cloned into pCR2.1-TOPO vector (Invitrogen) per the manufacturer's instructions. After confirming the sequence, the product was subcloned between KpnI and XhoI sites of the pGL2-Basic Vector (Promega). To mutate the LEF1/TCF4 binding site on the *HOTAIR* promoter, a mutagenesis primer was synthesized and the mutagenesis was performed using the QuikChange Multi Site-Directed Mutagenesis kit (Agilent Technologies) according to the manufacturer's instructions and the mutated clones were verified by sequencing. To measure the reporter activity, cells were plated in 24-well plates at a cell number of 6 × 10^4^ cells/well a day prior to the co-transfection with 0–0.8 μg of β-catenin/S37A cDNA or the control vector (pcDNA3), along with 50 ng HOTAIR-luciferase and 5 ng Renilla cDNA using Lipofectamine 2000 following the manufacturer's instruction (Invitrogen). For viability assay, cells were plated in 6-well plates at cell number of 5 × 10^5^ cells/well a day prior to the co-transfection with 4 μg of β-catenin/S37A cDNA or the control vector, along with 50 ng CMV-luciferase cDNA using lipofectamine 2000 following the manufacturer's instruction (Invitrogen). After 6 hr incubation, co-transfectant was split to 24-well plate at cell number of 5 × 10^4^. After 18 hr, another transfection was performed with 30 nmole of siScramble or siHOTAIR. Next day, cells were treated with 10 uM lapatinib, 10 μM iImatinib, the combination, or mock treated for 48 hr. The luciferase reporter activity was then measured by a GloMax 20/20 Luminometer (Promega).

### Quantitative RT-PCR (qRT-PCR)

Total RNA was extracted with Trizol (Invitrogen), and treated with DNase (Thermo Scientific). Reverse-transcription was performed with a cDNA Reverse Transcription Kit (Applied Biosystems). For real-time PCR, target RNA was amplified with gene-specific primers (see below) using the FastStart SYBR Green kit (Roche) on a 7900HT Fast Real-Time PCR System (Applied Biosystems). PCR reaction was initiated by heat activation of the FastStart Taq DNA polymerase at 95°C for 10 min, followed by 40 cycles of 95°C for 15 sec, 58°C for 30 sec, and 72°C for 60 sec. Relative levels were calculated using the comparative C_T_ method. Data were normalized to 18S or actin [44].

### Primers for qRT-PCR

18S-F: AGGATCCATTGGAGGGCAAGT

18S-R: TCCAACTACGAGCTTTTTAACTGCA

Actin-F: CTTCCCCTCCATCGTGGG

Actin-R: GTGGTACGGCCAGAGGCG

Myc-F: CGTCTCCACACATCAGCACAA

Myc-R: CACTGTCCAACTTGACCCTCTTG

HOTAIR-F: GGTAGAAAAAGCAACCACGAAGC

HOTAIR-R: ACATAAACCTCTGTCTGTGAGTGCC

BC200-F: TGGCTCACGCCTGTAATCC

BC200-R: CCCAGGCAGGTCTCGAACT

MALAT1-F: GACGGAGGTTGAGATGAAGC

MALAT1-R: ATTCGGGGCTCTGTAGTCCT

GAPDH-F: GTGAAGGTCGGTGTGAACGG

GAPDH-R: GATGCAGGGATGATGTTCTG

### Primers for ChIP of β-catenin

HOTAIR-TCF4-F: TGGCTTTAGCTCCTACATTAAG

HOTAIR-TCF4-R: CTGGAACAGATCCCAAACAA

### Primers for amplification of the *HOTAIR* promoter

HOTAIR(−1000–0)-F-KpnI aaggtaccgtccc acacatggacgctctc

HOTAIR(−1000–0)-R-Xhol aactcgaggcaggc agaaggcagggcctg

### Primer for site-directed mutagenesis of the TCF4/LEF1 site on the HOTAIR promoter

CCTAGTCCTCCTGATCCCCGTGAGCTCGCG GCAT

### Primers for constructing shRNA of *HOTAIR* and the control with scramble sequences

shHOTAIR-F: TCGAGGAACGGGAGTACAGAG AGATTTTCAAGAGAAATCTCTCTGTACTCCCGTTCG

shHOTAIR-R: AATTCGAACGGGAGTACAGAGA GATTTCTCTTGAAAATCTCTCTGTACTCCCGTTCC

shScramble-F: TCGAGCCTAAGGTTAAGTCGCC CTCGCTCTTCAAGAGAGAGCGAGGGCGACTTAAC CTTAGGG

shScramble-R: AATTCCCTAAGGTTAAGTCGC CCTCGCTCTCTCTTGAAGAGCGAGGGCGACTTAA CCTTAGGC

## SUPPLEMENTARY FIGURES AND TABLE


