# Supplementary material for: Combined inhibition of EGFR and c-ABL suppresses the growth of triple-negative breast cancer growth through inhibition of HOTAIR
Source: Oncotarget. 2015 Mar 10;6(13):11150–61. doi: 10.18632/oncotarget.3441 (PMC4484446; doi:10.18632/oncotarget.3441)
Supplement: Supplementary file 1 [file oncotarget-06-11150-s001.pdf]

SUPPLEMENTARY FIGURES AND TABLE

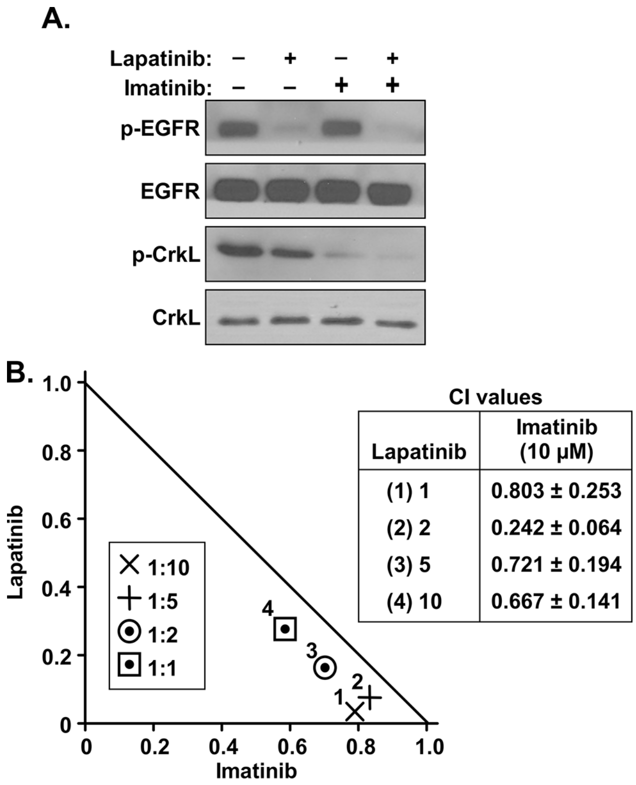

Supplementary Figure S1: Synergistic growth suppression by lapatinib and imatinib combination. **(A)** MDA-MB-231 cells were treated with lapatinib (10 μM), imatinib (10 μM), or both for 72 hours. Western analysis of total cell lysates showed that phospho-Y1068-EGFR and phospho-Y207-CrkL were inhibited by lapatinib and imatinib, respectively. **(B)** Isobologram based on dose-response curves for lapatinib and imatinib. Data points below the line represent synergistic drug interactions; points on the line indicate an additive interaction; points above the line indicate antagonism. The table shows the combination indexes (CI) of different doses of lapatinib with 10 μM of imatinib derived from three independent experiments. A combination index (CI) value of one indicates an additive interaction; a CI value greater than one indicates antagonism; and a CI value less than one indicates synergism.

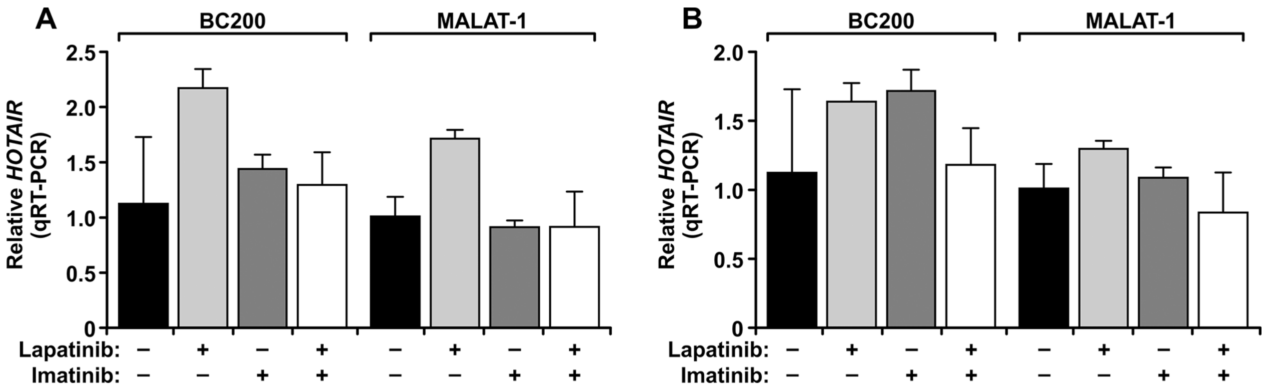

Supplementary Figure S2: Expression of lncRNA BC200 and *MALAT-1* does not respond to the dual treatment. MDA-MB-231 **(A)** and SUM159 **(B)** cells were untreated or treated with lapatinib, imatinib, or lapatinib plus imatinib as indicated. The levels of *BC200* and *MALAT-1* were then determined by qRT-PCR and normalized with *GAPDH* and *18S*. There is no significant statistic difference of RNA expression comparing the no treatment control and the dual treatment ($P > 0.05$).

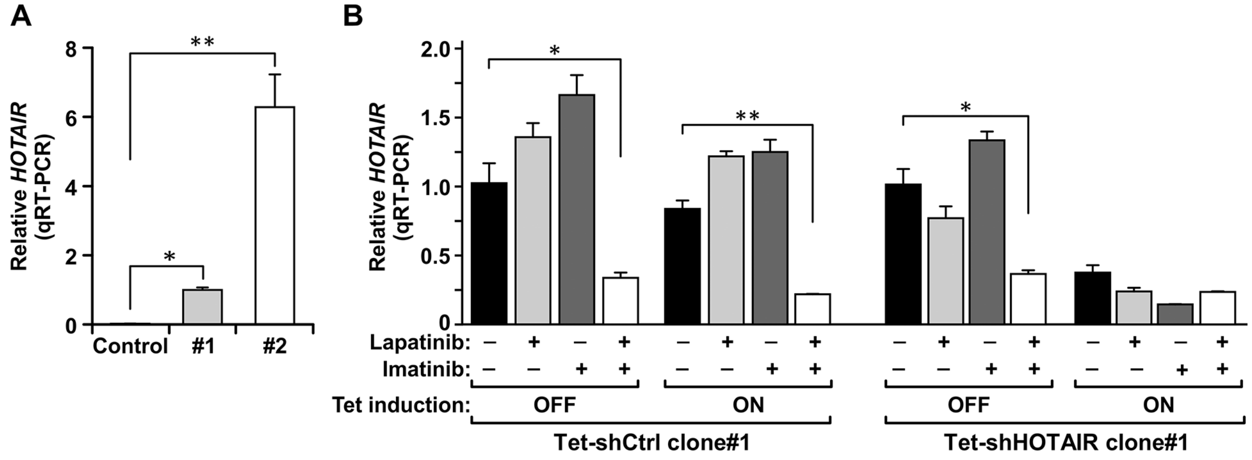

Supplementary Figure S3: *HOTAIR* expression in Figures 2B and 2C measured by qRT-PCR. (A) Relative *HOTAIR* expression in the stable clones of MDA-MB-231 used in Figure 2B. **(B)** Relative *HOTAIR* expression in the inducible clones used in Figure 2C. The qRT-PCR levels were normalized by 18S expression. *, $P < 0.05$; **, $P < 0.01$.

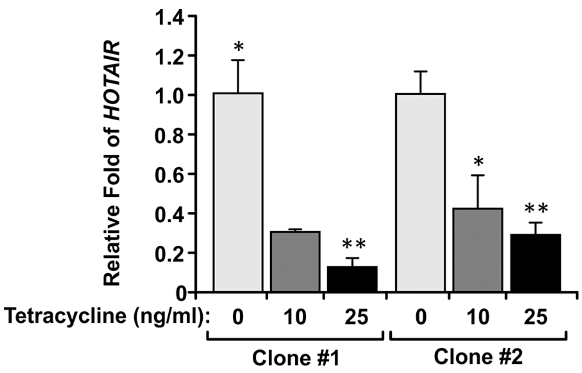

Supplementary Figure S4: *HOTAIR* expression in inducible *shHOTAIR* transfectants. MDA-MB-231 cells were engineered to expression shRNA of *HOTAIR* or the control shRNA of scrambled sequence under the control of the tetracycline-inducible promoter. Cells were induced by tetracycline of 10 ng/ml or 25 ng/ml for 96 hrs. The levels of *HOTAIR* were determined by qRT-PCR and normalized to *18S*. *, $P < 0.05$; **, $P < 0.01$.

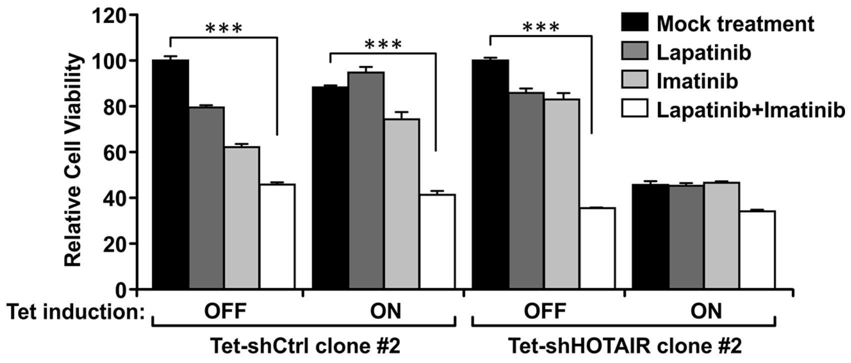

Supplementary Figure S5: Dual treatment inhibits cell growth through targeting *HOTAIR*. In addition to the clones shown in Figure 2C, an additional pair of MDA-MB-231-derived clones of tet-shHOTAIR and tet-shCtrl were induced by tetracycline for 96 hrs then treated with indicated drugs for 48 hrs. Viable cells were then measured by MTT assays. ***, $P < 0.005$.

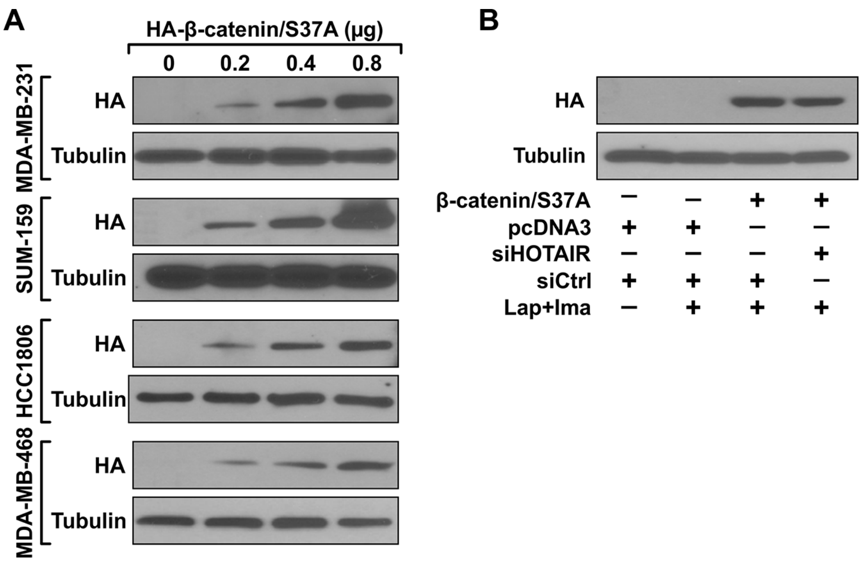

Supplementary Figure S6: Verification of β-catenin expression. (A) Expression of transfected HA-tagged β-catenin is determined by Western analysis using an anti-HA antibody for each indicated cell line treated separately but with the same conditions as described in Figure 3B. **(B)** Western analysis of HA-β-catenin expression in MDA-MB-231 cells treated separately but with the same conditions as described in Figure 3F.

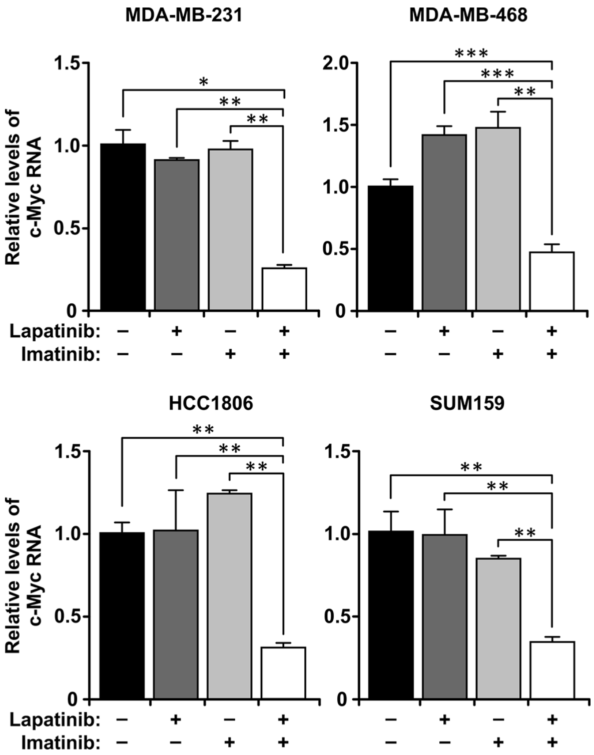

Supplementary Figure S7: Dual treatment suppresses c-Myc expression. MDA-MB-231 and MDA-MB-468 and SUM159 cells were mock treated, or treated with lapatinib alone (10 μM), imatinib alone (10 μM), or their combination for 48 hours. HCC1806 cells were mock treated or treated with lapatinib alone (5 μM), imatinib alone (10 μM), or their combination for 48 hours. The levels of c-Myc RNA were determined by qRT-PCR and normalized to 18S. *, $P < 0.05$; **, $P < 0.01$; ***, $P < 0.001$.

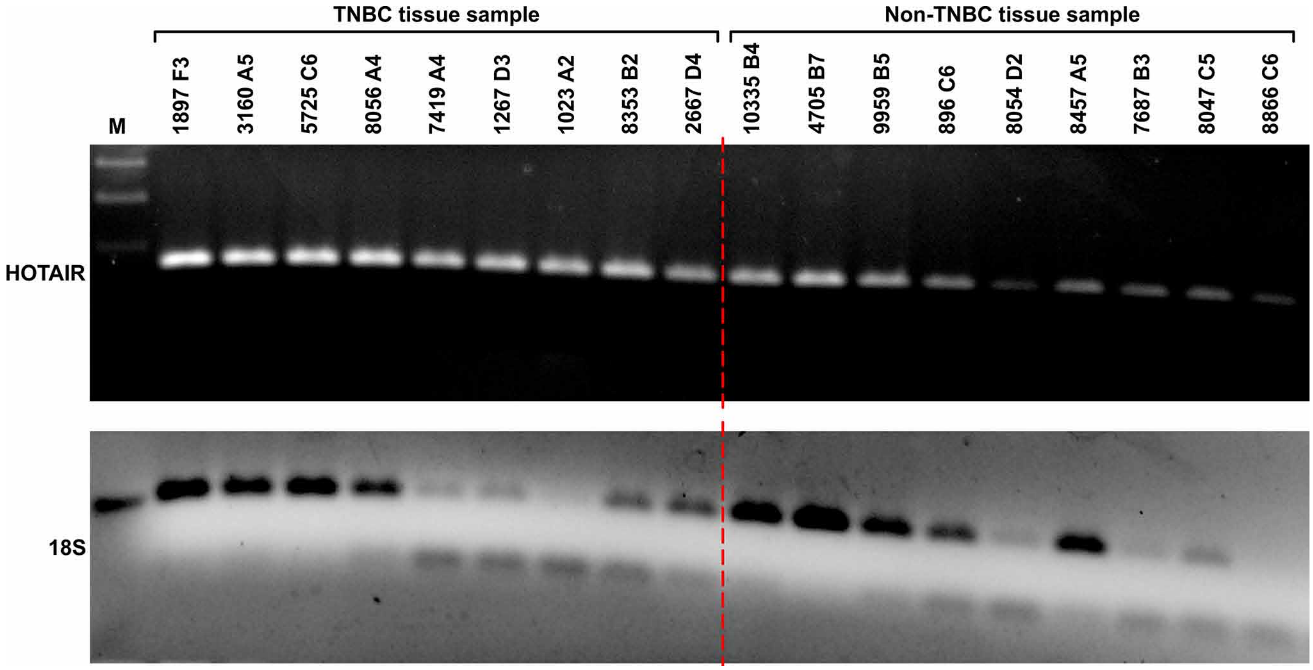

Supplementary Figure S8: Expression of *HOTAIR* is associated with TNBC. RNA samples from primary breast cancer tumor tissues of TNBC and non-TNBC were extracted following the instruction of the manufacturer (Zymo; Irvine, CA) and the levels of *HOTAIR* were determined by RT-PCR. 18S was shown as the input control.

Supplementary Table S1: Statistical analysis of Figure 1B

Weeks	Ctl vs Dual		Lap vs Dual		Ima vs Dual	
	Significance	*P* value	Significance	*P* value	Significance	*P* value
1	-	0.903099	-	0.885183	-	0.971655
4	-	0.605719	-	0.94504	-	0.935906
7	-	0.365714	-	0.777454	-	0.618999
10	-	0.320924	-	0.786416	-	0.551658
14	-	0.0721446	-	0.47566	-	0.368611
18	*	0.0130629	-	0.474232	-	0.250883
24	**	0.00950806	-	0.231792	-	0.0883949
27	***	0.00371837	-	0.0624445	*	0.0450856
30	***	0.00209735	*	0.0439888	***	0.00210895
33	***	2.0618E-05	*	0.0198929	***	6.7274E-05
37	***	3.1428E-11	*	0.0161623	***	2.2679E-05

*, $P < 0.05$
**, $P < 0.01$
***, $P < 0.005$
